# Alternative splicing of c-*fos *pre-mRNA: contribution of the rates of synthesis and degradation to the copy number of each transcript isoform and detection of a truncated c-Fos immunoreactive species

**DOI:** 10.1186/1471-2199-8-83

**Published:** 2007-09-21

**Authors:** Juan Jurado, Carlos A Fuentes-Almagro, María J Prieto-Álamo, Carmen Pueyo

**Affiliations:** 1 Universidad de Córdoba, Departamento de Bioquímica y Biología Molecular, Campus Rabanales, Edificio Severo Ochoa, planta-2^a^, 14071-Córdoba, Spain

## Abstract

**Background:**

Alternative splicing is a widespread mechanism of gene expression regulation. Previous analyses based on conventional RT-PCR reported the presence of an unspliced c-*fos *transcript in several mammalian systems. Compared to the well-defined knowledge on the alternative splicing of *fosB*, the physiological relevance of the unspliced c-*fos *transcript in regulating c-*fos *expression remains largely unknown. This work aimed to investigate the functional significance of the alternative splicing c-*fos *pre-mRNA.

**Results:**

A set of primers was designed to demonstrate that, whereas introns 1 and 2 are regularly spliced from primary c-*fos *transcript, intron 3 remains unspliced in part of total transcript molecules. Here, the two species are referred to as c-*fos*-2 (+ intron 3) and spliced c-*fos *(- intron 3) transcripts. Then, we used a quantitatively rigorous approach based on real-time PCR to provide, for the first time, the actual steady-state copy numbers of the two c-*fos *transcripts. We tested how the mouse-organ context and mouse-gestational age, the synthesis and turnover rates of the investigated transcripts, and the serum stimulation of quiescent cells modulate their absolute-expression profiles. Intron 3 generates an in-frame premature termination codon that predicts the synthesis of a truncated c-Fos protein. This prediction was evaluated by immunoaffinity chromatography purification of c-Fos proteins.

**Conclusion:**

We demonstrate that: (i) The c-*fos*-2 transcript is ubiquitously synthesized either *in vivo *or *in vitro*, in amounts that are higher or similar to those of mRNAs coding for other Fos family members, like FosB, ΔFosB, Fra-1 or Fra-2. (ii) Intron 3 confers to c-*fos*-2 an outstanding destabilizing effect of about 6-fold. (iii) Major determinant of c-*fos*-2 steady-state levels in cultured cells is its remarkably high rate of synthesis. (iv) Rapid changes in the synthesis and/or degradation rates of both *c-fos *transcripts in serum-stimulated cells give rise to rapid and transient changes in their relative proportions. Taken as a whole, these findings suggest a co-ordinated fine-tune of the two *c-fos *transcript species, supporting the notion that the alternative processing of the precursor mRNA might be physiologically relevant. Moreover, we detected a c-Fos immunoreactive species corresponding in mobility to the predicted truncated variant.

## Background

Activator protein-1 (AP-1) is a dimeric transcription factor regulating major physiological processes such as cell proliferation, differentiation, neoplastic transformation, apoptosis, and response to stress [1]. Main AP-1 components in mammals are members of the Fos and Jun protein families. The Fos family includes the products of the c-*fos *(the cellular counterpart of oncogene v-*fos*), *fosB*, *fra-1 *and *fra-2 *genes. Fos proteins associate with Jun, but also with other basic leucine-zipper (bZIP) proteins to create a variety of AP-1 complexes [2].

The expression of c-*fos *is subjected to a tight regulation at multiple levels. The c-*fos *gene undergoes rapid and transient transcriptional activation in response to a variety of extracellular stimuli [3]. Both c-*fos *mRNA and protein turn over with short half-lives [4,5]. Additionally, when the c-Fos protein is over-synthesized, the c-*fos *gene is transcriptionally repressed [6].

Alternative splicing is a widespread mechanism of gene expression regulation. The typical form of this regulation results from tissue- or temporal-specific splicing events that lead to the synthesis of either productive (protein-coding) or non-productive (no protein- coding) RNAs. Regarding transcription factors, alternative splicing preferentially adds or deletes domains that are important for their architectures and functions ([7] and references herein). The production of multiple isoforms is a common strategy for regulating the activity of genes within the Fos family (e.g. [8]). A particularly instructive example is the differential splicing of the *fosB *transcript, which generates two mRNAs that encode proteins with antagonistic activities. Briefly, alternative splicing removes codons 238–284 and causes a shift in the reading frame that places a stop codon following the bZIP region. The truncated protein (referred to as ΔFosB), missing the C-terminal 101 aa of FosB, functions as a trans-negative regulator of transcriptional activation and transformation, presumably by competing with the full-length Fos protein in the dimerization with Jun and binding to DNA steps [9-11].

Previous analyses [12,13] based on RT of total RNA followed by conventional PCR have reported the presence of a transcript isoform (hereinafter referred to as c-*fos*-2) in several mammalian systems: (i) rat brain following kainic acid treatment, (ii) primary cortical culture of mouse brain treated with lipopolysaccharide, (iii) cartilage from newborn mouse, and (iv) mouse and human cultured stromal cells. Compared to the well-defined knowledge on the alternative splicing of *fosB*, the physiological relevance of c-*fos*-2 transcript in regulating c-*fos *expression has not been well addressed.

The work described herein aimed to gain further insights into the functional significance of the alternative splicing of c-*fos*. To this end, we first undertook a systematic and meticulous quantitation of the real copy numbers of c-*fos *transcript variants by means of a real-time PCR methodology [14-17]. We tested how the organ context, gestational age, synthesis and turnover rates, and serum stimulation of quiescent cells modulate the absolute expression profiles of the investigated c-*fos *transcripts. The translation of these transcripts predicts the synthesis of a truncated c-Fos protein, in addition to the canonical full-length counterpart. From cultured cells we partially purified a c-Fos immunoreactive species corresponding in mobility to the predicted truncated species.

## Results

### Alternative splicing variants of c-*fos *messenger

**Figure 1:**
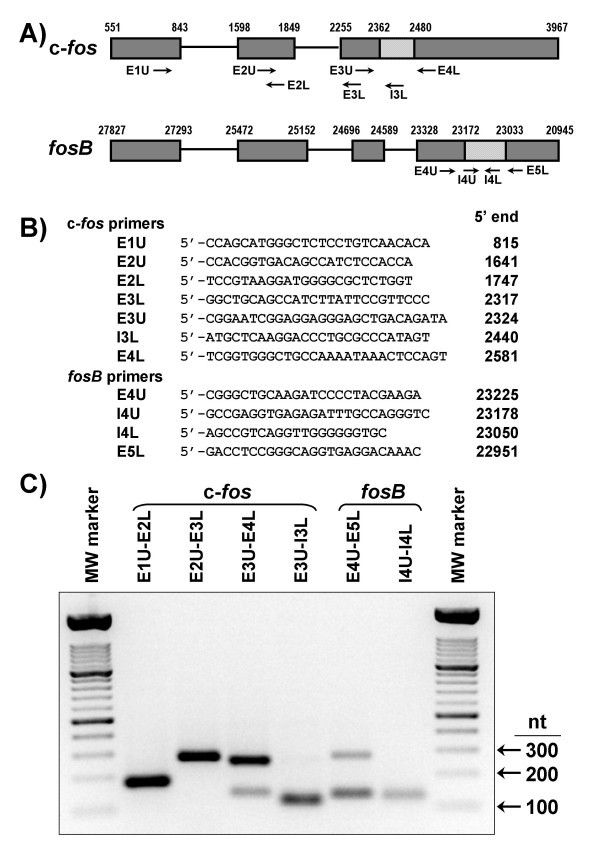
**RT-PCR amplicons from c-fos and fosB transcripts**. A) Schematic drawing of c-*fos *and *fosB *gene structures. Exons (E) are boxed and shaded in grey. Introns (I) are indicated by lines between exons, except for the alternatively spliced sequences that are indicated by brick filled in boxes. The beginning and end of exons are indicated by the corresponding nucleotide positions (NCBI/GeneBank accession numbers: V00727 and AF093624 for c-*fos *and *fosB*, respectively). B) Names, sequences and 5'-position of upper (U) and lower (L) primers. C) Agarose (1.5%) gel electrophoresis analysis of RT-PCR products generated by different primer pairs (identified at the top of each line). Total RNA from NIH 3T3 cells was used as template. Forty cycles of PCR were performed as detailed [16]. Genomic DNA was further amplified by the E1U-E2L primer pair to exclude the possibility that our PCR conditions were not optimal for the amplification of the longest theoretical fragment, i.e. the 933 nt amplicon that should be observed if intron 1 remained unspliced in the c-*fos *transcript population [see Additional file 7]. The molecular weight marker was a 100-nt ladder from Roche.

Differential pre-mRNA splicing is an important mode of regulating the steady-state abundance of a specific mRNA. Based on the known structure of c-*fos *gene (Fig. 1A), a set of primers (Fig. 1B) was designed to investigate the occurrence of any splicing variant that might be derived from the primary c-*fos *transcript. Gene *fosB *was included for comparison. Primer pairs E1U-E2L and E2U-E3L (located in exons 1 and 2 and in exons 2 and 3, respectively) generated single RT-PCR amplicons of expected sizes and sequences after regular splicing of introns 1 and 2 (Fig. 1C). However, the primer pair E3U-E4L revealed the presence of two transcript populations: while the shorter amplicon (141 nt) corresponded to the canonical spliced form of c-*fos *message, the larger amplicon (258 nt) retained the 117 nt of intron 3. These data confirmed that intron 3 is not always spliced from the primary c-*fos *transcript [12,13]. To quantitate this second transcript species, an additional primer pair (E3U-I3L) was designed. As expected, these primers gave a unique RT-PCR product of the predicted size (120 nt) and sequence.

### Steady-state molecules of c-*fos *transcripts in animal tissues and cultured cells

Here we used a quantitatively rigorous approach based on RT and real-time PCR amplification [14-17] to provide novel information on the actual steady-state copy numbers of the two c-*fos *transcripts. Quantitations were carried out in adult mouse organs, whole mouse embryos at different developmental stages, and murine cultured cells.

**Figure 2:**
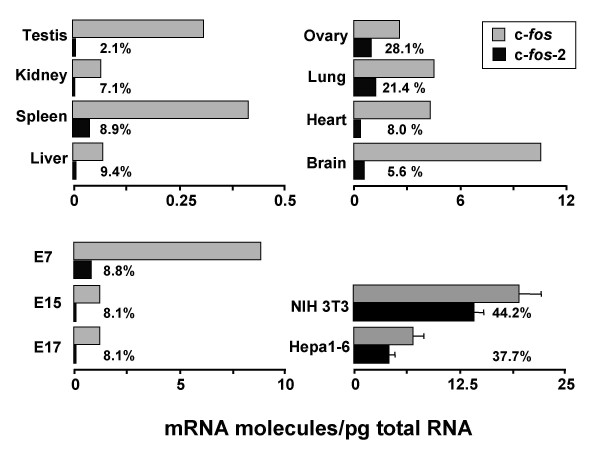
**Organ-, embryo-, and cell type-associated differences in basal amounts of c-fos transcripts**. Data are the numbers of transcript molecules per pg of total RNA. Regarding cultured cells, error bars (SEM) indicate transcript level variability among 13 (NIH 3T3) or 8 (Hepa1-6) independent cultures. Total RNA (pool of ~200 BALB/c mice) from animal organs and whole embryos were from Clontech. No error bars are shown for quantitations of these commercial samples of total RNA. E17 embryos are in late gestation (total of about 19 days). The percentage of c-*fos*-2 in total transcript amount (c-*fos*-2 plus spliced c-*fos*) is indicated for each of the sample examined.

We found that both transcripts were ubiquitously expressed and presented vast differences in abundance depending on the sample (Fig. 2). In the adult organs examined, the canonical spliced c-*fos *mRNA displayed high steady-state levels in brain (10 molecules/pg of total RNA) followed by heart, lung, and ovary (average of 4 molecules/pg), as compared to spleen and testis (about 0.4 molecules/pg), and the rest of the organs (around 0.07 molecules/pg). Notably, this quantitative tissue-expression profile is not in good agreement with qualitative RT-PCR results in public database. For instance, qualitative RT-PCR (accession ID, MGI:1204456) generated a strong c-*fos *amplification band from either liver or lung RNA and no detectable signal from kidney RNA. However, quantitative data given here (Fig. 2) showed much lower expression levels in both liver and kidney (around 0.07 molecules/pg RNA) than in lung (5.7 molecules/pg RNA). These discrepancies might be due, at least partly, to the experimental uncertainties of the end-point RT-PCR methodology.

The c-*fos*-2 variant was present in smaller amounts in all 8-tissue samples, where its presence accounted for different percentages of the total transcript molecules, ranging from more than 20% in lung and ovary to about 2% in testis. Regarding whole embryos, an over 7-fold overexpression in both c-*fos *transcripts occurred as early as E7 (gastrulating embryos). Thereafter, their amounts declined to approximately the average numbers of the steady-state copies measured in adult tissues. Therefore, the c-*fos*-2 species accounted for a constant 8–9% of the total transcript molecules throughout mouse embryonic development.

Elevated levels of the two c-*fos *transcripts were also quantitated in NIH 3T3 and Hepa1-6 confluent cells. Interestingly, at least in these 2 cell lines, we observed both products with an about equal yield. This finding differs from a recent report in which confluent stromal cells expressed only the c-*fos*-2 transcript [13]. With regard to the absolute amounts of transcripts coding for the rest of proteins within the Fos family (Table 1 and data not shown), we found that the c-*fos-2 *variant was nearly as abundant in lung, liver, and kidney and in NIH 3T3 cells as the transcripts coding for Fra-1 and Fra-2. Comparatively, the transcript coding for FosB displayed a much lower level.

**Table 1:** Absolute steady-state levels of transcripts encoding proteins of the Fos family in mouse lung

mouse	*c-fos*	*c-fos-2*	*fosB*	*fra1*	*fra2*
1	2.80	0.59	0.035	0.96	1.93
2	4.52	0.62	0.039	0.98	2.42
3	3.37	0.81	0.033	1.03	2.48
4	3.04	0.66	0.030	1.03	1.63
5	2.00	0.49	0.022	1.15	2.73
6	3.08	0.47	0.023	1.26	2.34
7	2.14	0.16	0.012	1.26	2.04
8	2.41	0.41	0.018	1.44	2.60
9	3.57	0.61	0.022	1.51	2.61
10	3.71	0.88	0.033	1.29	2.82
11	4.16	0.42	0.014	1.13	2.67
12	3.90	0.58	0.038	1.37	2.94
mean ± SEM	3.22 ± 0.23	0.56 ± 0.053	0.027 ± 0.003	1.20 ± 0.050	2.44 ± 0.11

Male BALB/c mice of 7 weeks of age were obtained from Charles River Laboratories (Spain). Following a 3-day acclimation period animals were individually killed by cervical dislocation. The lung was removed and immediately frozen in liquid nitrogen. Mice were handled according to the norms stipulated by the European Community. The investigation was performed after approval by the Ethical Committee of the University of Córdoba (Spain). Data are transcript molecules/pg of total RNA.

### Differential stability of the two c-*fos *transcripts

**Figure 3:**
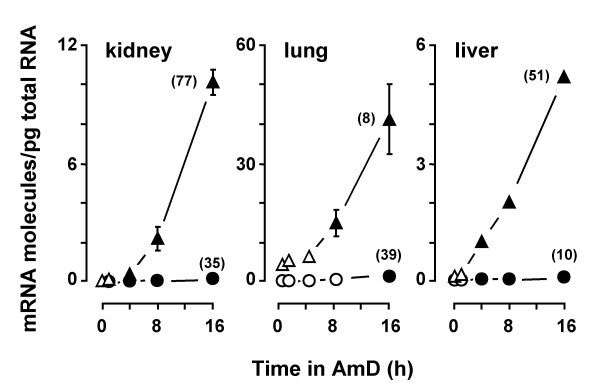
**Time-courses of c-fos transcripts in mouse kidney, lung and liver in response to AmD**. Male BALB/c mice (see legend of Table 1) were intraperitoneally injected with 2 mg kg^-1 ^body weight of AmD dissolved in phosphate-buffered saline. Animals injected with phosphate-buffered saline served as vehicle controls. Total RNA was extracted at the indicated times. Data are the means of c-*fos *(triangles) or c-*fos*-2 (circles) molecules/pg of total RNA ± SEM (n = 3 mice). Data at 0 min represent the mean values ± SEM of 5 control mice. No time-related effect was noted in these vehicle controls. Some error bars are not visible because of small standard errors. Statistical significance was evaluated using analysis of variance followed by *post hoc *multiple comparison according to the Student-Newman-Keuls method. Significant differences relative to control animals are indicated by filled-in symbols. For each transcript, the maximal fold increment is given in parentheses.

The intrinsic stability of any transcript is an important component of the gene expression program. We first tried to determine the half-life of each investigated c-*fos *transcript in a whole animal study. For this purpose, we administered actinomycin D (AmD) to stop transcription and then quantitated the number of transcript molecules remaining at various times after the transcription shut-off. Surprisingly, in contrast to previous results with other murine mRNAs [15], the decay rates of the c-*fos *transcripts could not be calculated in animals, since their hepatic, pulmonary and renal levels increased (not decreased) upon the intraperitoneal injection of AmD (Fig. 3). These results indicate that c-*fos *(an early response gene) is up-regulated *in vivo *by AmD under conditions in which the transcription of other genes is blocked [15]. We have previously addressed this problem in *Saccharomyces cerevisiae *[18], where the levels of mRNAs that are particularly responsive to environmental changes are up-regulated by current procedures for blocking transcription (i.e. RNA polymerase inhibition by specific drugs or thermal inactivation of a temperature-sensitive mutant). Since AmD doses higher than 2 mg kg^-1 ^body weight and incubation times longer than 16 h could not be used without affecting the apparent healthy aspect of mice, we next considered the possibility of quantifying the decay rates of the investigated c-*fos *transcripts in the cell-cultured model.

**Figure 4:**
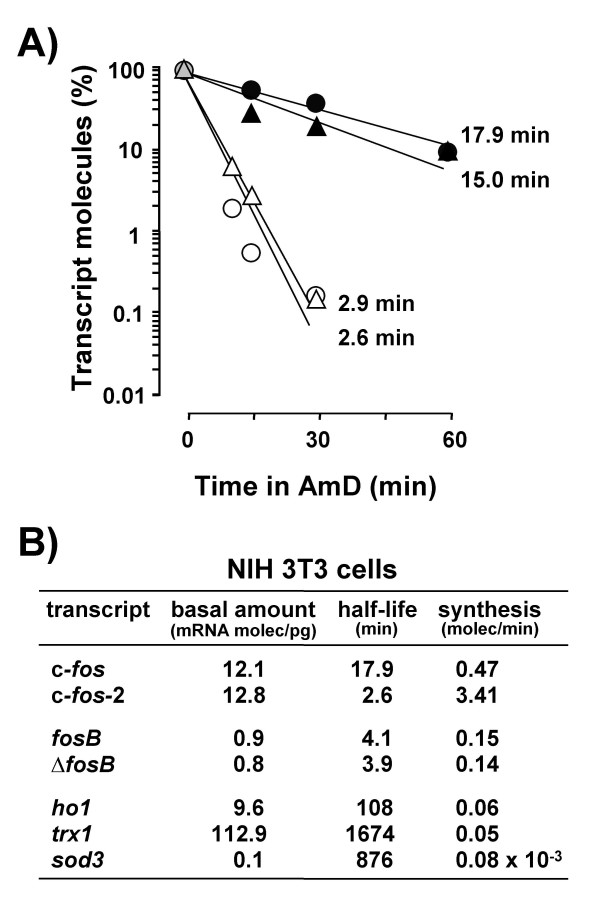
**Rates of transcript decay and synthesis in cultured cells**. A) Decay rates of the c-*fos*-2 (open symbols) and c-*fos *(solid symbols) transcripts in NIH 3T3 (circles) and Hepa1-6 (triangles) cells. Data are the percentages of transcript molecules remaining after the addition of AmD. The half-life (t_1/2_) values calculated from the resulting decay lines are given for comparisons. B) Rates of transcript decay and synthesis in NIH 3T3 cells. Transcript synthesis rates (in molecules/min) were calculated from the basal amounts of transcript molecules (per pg of total RNA) and their estimated half-lives (in minutes). These calculations are based on the assumption that though mRNA degradation is a complex process, it follows first-order kinetics[36]. The steady-state levels and rates of decay and synthesis of transcripts coding for both types of FosB protein (*fosB *and Δ*fosB*) and for heme oxygenase 1 (*ho1*), thioredoxin 1 (*trx1*) and superoxide dismutase 3 (*sod3*) are included for comparison.

In contrast to animals, in cultured cells the molecule number of each c-*fos *transcript isoform decayed exponentially in the presence of AmD and their half-lives were readily calculated from the equation of each decay line (Fig. 4A). The turnover of the two spliced forms of *fosB *mRNA and those of various non proto-oncogene transcripts were also determined for comparison (Fig. 4B). We found that the two species of c-*fos *transcripts displayed rather different half-lives in cultured cells: the c-*fos-*2 decayed with a half-life of less than 3 min, whereas the half-life of the spliced c-*fos *messenger was over 15 min (Fig. 4A). Moreover, the kinetics and magnitudes of the quantitated decays suggested that c-*fos*-2 is not merely a splicing intermediate in the production of the mature c-*fos *transcript [see theoretical considerations in Additional file 1]. In contrast to c-*fos*, both *fosB *transcripts exhibited identical half-lives of about 4 min (Fig. 4B). Transcript turnover rates in the order of minutes was a characteristic of proto-oncogenes since other mRNA species such as those encoding for heme oxygenase 1, thioredoxin 1, and superoxide dismutase 3 presented turnover rates in the order of hours.

The steady-state abundance of any transcript depends on the balance between two opposing factors, its rate of formation and degradation. We show that though both c-*fos *transcripts are similarly abundant in cultured cells, the c-*fos-*2 turned over about 6-fold faster than the spliced c-*fos *mRNA. Both findings can only be conciliated if, at the same time, the c-*fos-*2 isoform is produced at a higher rate. Having determined the numbers of transcript copies and the transcript half-lives, we can estimate their synthesis rates. The result of such a calculation (Fig. 4B) suggests that the major determinant of c-*fos-*2 transcript level in cultured cells is its high rate of synthesis, as compared to that of the spliced c-*fos *variant, and particularly to those of both *fosB *and of non proto-oncogenes mRNAs.

**Figure 5:**
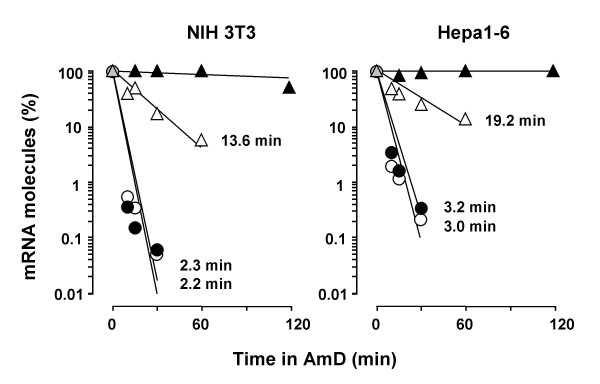
**Changes in transcript decay upon translation inhibition**. Decay rates of c-*fos*-2 (circles) and c-*fos *(triangles) transcripts were determined as in Fig. 4, excepting that half of the plates were pretreated for 15 min with Cx (10 μg/ml). Then, AmD was added to all the plates and transcript decay was followed in the presence (solid symbols) and absence (open symbols) of Cx.

Next, we asked ourselves whether cycloheximide (Cx), a protein synthesis inhibitor, leads to the stabilization of the alternative c-*fos*-2 transcript. We found (Fig. 5), that the fast decay rate of the c-*fos*-2 species is not coupled to translation, since it went on being ≤ 3 minutes in the presence of Cx, irrespective of the cell type (NIH 3T3 or Hepa1-6). In contrast, as expected [5], the inhibition of the translation transformed the spliced c-*fos *mRNA into a stable species (at least in the 120 min period examined).

### Transcript levels upon serum stimulation

Previous studies have shown that the stimulation of quiescent cells with 15% CS causes a rapid transient increase in c-*fos *mRNA [19]. To gain more insight into the mechanisms that might be influencing the cellular level of the alternative c-*fos*-2 transcript, we decided to analyse whether its accumulation mode after serum induction was identical or not to that of the canonical spliced mRNA.

**Figure 6:**
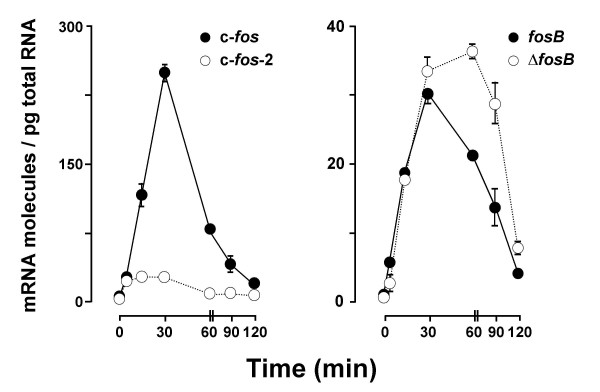
**Time-course of c-fos (left) and fosB (right) transcript levels in serum-stimulated NIH 3T3 cells**. Data are the means of transcript molecules per pg of total RNA ± SEM from independent cultures. Some error bars are not visible because of small SEM.

We found (Fig. 6) that CS induced different expression profiles for both the spliced c-*fos *and the c-*fos*-2 transcript variant. In both cases, a dramatic 11-fold increase (from about 2.3 to 25 molecules/pg) was quantitated as soon as 5 min after serum addition. However, following this early up-regulation, while the c-*fos*-2 copy number remained relatively constant within 5–30 min, the amount of the spliced c-*fos *mRNA increased until a maximum of 100-fold was reached 30 min past stimulation. Thereafter, both transcript levels decayed with different kinetics: spliced c-*fos *mRNA molecules decreased to 50% of maximum in 20 min, while c-*fos-*2 lasted 37 min. Overall, these distinguishing features caused rapid changes in the relative abundance of both transcripts throughout the period analyzed in this experiment. Comparatively, our quantitative PCR analysis revealed a much less differential expression of both *fosB *mRNA isoforms. Moreover, in contrast to previous observations [11], the expression of *fosB *did not precede that of Δ*fosB *mRNA at any time following serum stimulation. In fact, both transcripts were induced in a close parallelism throughout the first 30 min of serum treatment, and, additionally, the Δ*fosB *mRNA increase persisted longer (Fig. 6).

Rapid changes in the rate of synthesis and/or the rate of degradation of either the canonical c-*fos *mRNA or the c-*fos*-2 variant, or both, would give rise to rapid changes in the relative magnitudes of these transcripts. We examined this possibility by calculating the formation and degradation rates of both transcripts in quiescent and serum-stimulated cells. As shown in Table 2, the half-life of the c-*fos*-2 variant remained essentially identical to that seen for this transcript in confluent cells. Therefore, serum-related changes in the copy number of the c-*fos*-2 transcript were entirely explained by differences in its rate of synthesis. Nonetheless, two trends could be envisaged regarding the spliced c-*fos *mRNA: while an outstanding induction of its synthesis would be the only determinant for the large up-regulation of spliced c-*fos *mRNA within the first 30 minutes after serum addition, the subsequent rapid return of this transcript to the prestimulation levels would require an accelerated turnover rate (see Fig. 6).

**Table 2:** Rates of synthesis and degradation of c-*fos *and c-*fos*-2 transcripts upon serum stimulation

	c-*fos*			c-*fos*-2		
		
NIH 3T3 cells	amount (molec/pg)	half-life (min)	synthesis (molec/min)	amount (molec/pg)	half-life (min)	synthesis (molec/min)
confluent	12.1	17.9	0.47	12.8	2.6	3.41
quiescent	2.6	17.5	0.10	2.2	2.3	0.66
serum (15 min)	116.1	17.1	4.71	28.1	2.3	8.47
serum (60 min)	77.1	8.5	6.29	10.2	2.7	2.62

Experimental conditions are as in Figs. 4 and 6. Confluent cell data are those in Fig. 4.

### Translation of c-*fos*-2 transcript

**Figure 7:**
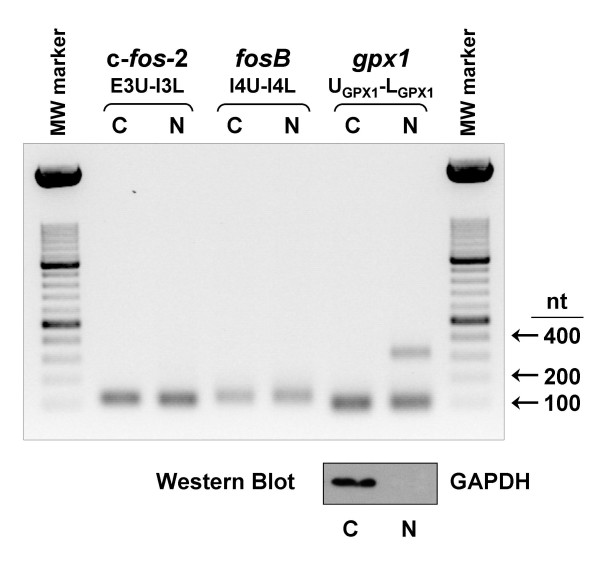
**RT-PCR analysis of cytoplasmic and nuclear RNAs**. Cytoplasmic (C) and nuclear (N) RNAs were isolated by using the PARIS™ Kit (Ambion) according to the manufacturer's protocol. RT-PCR conditions were as in Fig. 1. The RT-PCR product of 120 nt amplified by the E3U-I3L primer pair (see Fig. 1) indicates the presence of the c-*fos*-2 transcript in both cytoplasmic and nuclear fractions. Amplification of the unspliced *fosB *transcript (I4U-I4L primers) was included for comparison. Transcript coding for glutathione peroxidase 1 was amplified as control of efficient cellular fractionation. Primer U_GPX1 _(5'-GCAGAAGCGTCTGGGACCTCGTG) was located in *gpx1 *exon 1, and primer L_GPX1 _(5'-GGGAATTCAGAATCTCTTCATTCT TGCCA) in *gpx1 *exon 2 (the intron between both exons is 218 nt long). U_GPX1_- L_GPX1 _primers generated a single RT-PCR product of 101 nt when using the cytoplasmic RNA as template, indicating no detectable contamination with nuclear *gpx1 *pre-mRNA. In contrast when using the nuclear RNA as template, a fragment 319 nt long was detected in addition to the expected product from spliced *gpx1 *mRNA. Since the Ambion's PARIS™ Kit is designed for the isolation of both RNA and protein from the same sample, efficient cellular fractionation was further confirmed by Western blot analysis (botton panel) with an Ab specific for the cytoplasmic glyceraldehyde-3-phosphate-dehydrogenase (GAPDH). GAPDH was present only in the cytoplasmic fraction, further indicating no detectable contamination with the nuclear fraction.

Intron 3 generates in c-*fos-*2 transcript an in-frame translation premature termination codon (PTC), thereby predicting (if translated) the synthesis of a C-terminally truncated protein 169 aa long. In an attempt to obtain experimental proof of the synthesis of the predicted truncated c-Fos protein, we first investigated whether the c-*fos*-2 transcript was present in the cellular cytoplasm. To this end, RNA was isolated from separate cytoplasmic and nuclear fractions of NIH 3T3 cells, and then the resulting RNAs were analyzed by end-point RT-PCR. As shown in Fig. 7, the c-*fos*-2 transcript was present in both cellular fractions, like the unspliced *fosB *transcript. In contrast, no unspliced *gpx1 *pre-mRNA was present in the cytoplasmic fraction showing an efficient cellular fractionation.

**Figure 8:**
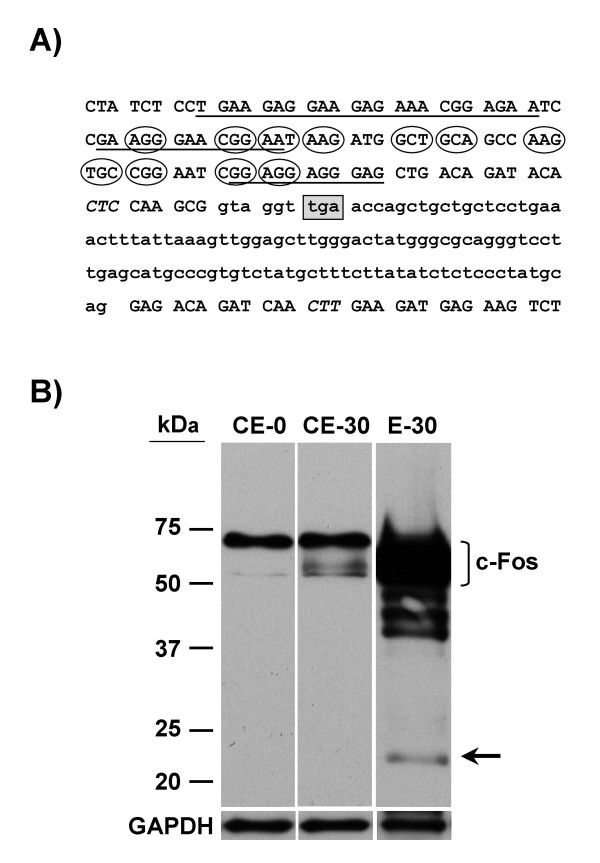
**Detection of c-Fos immunoreactive species**. A) Nucleotide sequence of c-*fos *intron 3 and flanking exons 3 and 4. Lower case letters refer to intronic nucleotides and upper case letters to exonic nucleotides. Minimal functional sequences within the mCRD are underlined. The encircled triplets encode the aa residues that contact to DNA. Triplets encoding two out of the five-leucine residues of the LZ motif are indicated in italics. The stop codon in intron 3 is boxed and shaded in grey. B) Western blot analysis of c-Fos proteins purified by immunoaffinity chromatography. A crude extract from NIH 3T3 cells stimulated with serum for 30 min was loaded onto the immunoaffinity column. The column eluate (E-30), as well as the input crude extract (CE-30) and the crude extract from serum-starved (CE-0) cells were subjected to inmunoblot analysis as described under "Methods". The positions of molecular size standards are indicated on the left in kDa. GAPDH was used for loading control.

Given the presence of the c-*fos*-2 transcript in the cytoplasm, we next evaluated whether the truncated c-Fos variant accumulated in serum-stimulated cells. For this purpose, c-Fos proteins were partially purified by immunoaffinity chromatography from NIH 3T3 cell extract following 30 min serum stimulation. Immunoblot analysis with anti-cFos Ab of the eluate column revealed several electrophoretically distinguishable bands (Fig. 8B). The higher molecular weight bands matched the multiple forms of the full-length c-Fos protein that appeared in the range of 53- to 68-kDa [4,20]. Bands detected in the range of 42- to 50-kDa might be c-Fos breakdown products. Finally, the discrete immunoreactive species corresponding in mobility to a ~23-kDa protein would be the truncated c-Fos form. This band was not detected in crude extract from either serum-starved or serum-stimulated cells. The possibility that the ~23-kDa band would be primary Ab light chains leaching off the immunoaffinity column was excluded by using only the secondary Ab [see Additional file 2]. Furthermore, the possibility that the ~23-kDa band would be a non-specific protein co-purified with the rabbit anti-cFos Ab was excluded by using a different primary Ab in the Western blotting [see Additional file 3].

## Discussion

c-Fos proto-oncoprotein is a key cell regulator, whose improper expression is oncogenic, both in cultured cells and living organisms. To avoid the deleterious effects of deregulated expression, c-*fos *is subjected to numerous transcriptional and posttranscriptional controls [2]. Differential pre-mRNA splicing is an important mode of regulating the activity of mammalian genes (e.g. [7]). Our interest in c-*fos *splicing stems from previous studies on *fosB *where a truncated spliced variant missing the C-terminus of FosB appears to be of great physiological relevance, e.g. in mediating long-term adaptive changes in the nervous system [21].

Murine c-*fos *gene comprises 4 exons and 3 introns spanning ~3.4 kb of chromosome 12. By means of specific primers, we first explored the presence of intron sequences in the transcript population derived from c-*fos*. We demonstrated that whereas introns 1 and 2 are regularly spliced from the precursor RNA, intron 3 remains unspliced in part of the total transcript molecules. Following this finding, we performed a robust and sensitive RT-PCR methodology for providing for the first time a comprehensive quantitation of the absolute expression levels of each transcript isoform: the canonical spliced c-*fos *and the alternative c-*fos*-2. Comparisons of expression levels across 8 mouse organs, 3 fetal stages and 2 cultured cell lines led to four observations: (i) The c-*fos*-2 transcript is ubiquitously synthesized either *in vivo *or *in vitro *situations. (ii) This isoform is present in amounts that are higher or similar to those of mRNAs coding for other Fos family members like FosB, ΔFosB, Fra-1 or Fra-2. (iii) The c-*fos*-2 variant is frequently processed in certain mouse tissues, like ovary and lung, and in confluent cell cultures, where both transcript isoforms are produced in about equal quantities. (iv) No obvious relationship exists between total amount of c-*fos *transcripts and the relative quantity of the c-*fos*-2 form. For instance, c-*fos*-2 accounted for about 9% of total transcript molecules in liver and about 6% in brain, though this last organ exceeds the hepatic steady-state amount by over 75-fold. On the whole, these findings support the notion that the c-*fos*-2 copy number might be controlled.

The number of copies of any transcript is determined by a delicate balance between opposing synthesis and degradation mechanisms. Here we show that the presence of intron 3 confers to the c-*fos*-2 transcript a destabilizing effect of about 6-fold, e.g. reducing the transcript half-life from 18 to 3 min in confluent NIH 3T3 cells (Fig. 4).

The canonical c-*fos *mRNA is targeted for decay by two functionally independent instability determinants. One of these determinants (designated mCRD) is located in the protein-coding region and the other (an AU-rich element) in the 3'-untranslated end of the c-*fos *mRNA [22]. The mRNA decay mediated by mCRD represents a "suicide" mechanism in which translation of the mCRD-containing RNA results in the rapid degradation of the message [23]. Here we show that the half-lives of the c-*fos *and c-*fos*-2 transcripts are affected differently by inhibition of the translation, since cycloheximide caused the stabilization of only the canonical c-*fos *mRNA (Fig. 5). This result suggests that the shorter half-life of the alternative c-*fos*-2 species is independent of translationally coupled mRNA turnover mechanisms, like the one directed by the mCRD determinant. Accordingly, the c-*fos*-2 transcript with the PTC lying in the last intron 3 (Fig. 8A) should be also immune to the so-called nonsense-mediated decay (NMD). It is noteworthy that this latter was anticipated given that one critical determinant of whether a mammalian transcript is subjected to NMD is the presence of a splicing-generated exon-exon junction >50–55 nt downstream the PTC (recently reviewed in [24] and [25]). In the absence of these two translationally coupled mRNA turnover mechanisms, an intriguing question remains to be answered: Why is the c-*fos*-2 transcript degraded at a faster rate than the canonical c-*fos *message?.

A recent paper by Moraes *et al *[26] indicates that the CUG-binding protein (CUG-BP) plays a role in c-*fos *mRNA decay. By means of an *in vitro *deadenylation assay, they showed that CUG-BP binding to c-*fos *mRNA stimulates the poly(A) shortening by the PARN deadenylase. Since deadenylation is a rate-limiting step in the turnover of most mRNAs, we hypothesized that the presence of intron 3 in the c-*fos*-2 transcript might accelerate its decay by the above-referred mechanism. We reasoned that in this case, the proportion between both c-*fos *transcript variants might change when using an anchored oligo(dT)primer (5'-T_20_VN-3'), rather than random primers, in the cDNA synthesis. This approach allowed us to track specifically the transcripts that retained poly(A)^+ ^tails of a sufficient length to allow priming. We found [see Additional file 4] that the poly(A)^+ ^population has practically the same copy numbers of c-*fos *and c-*fos*-2 transcripts as the overall RNA population. Moreover, the rate at which each transcript disappeared from the poly(A)^+ ^population was similar to their overall decay rates [see Additional file 4]. These data seem to favour the idea that the two transcripts undergo deadenylation at the same rate, which seems to be in agreement with the synchronous deadenylation pattern seen for c-*fos *RNA both *in vivo *and *in vitro *[26,27]. Further work should pursue the identification of new instability determinants within the c-*fos*-2 transcript variant.

Shur *et al *[13] have recently reported rapid changes in c-*fos*-2 and c-*fos *transcript levels in response of the osteoblastic cells to a challenge with dexamethasone (a pharmacological glucocorticoid hormone). Control of c-*fos *splicing through the interaction of the glucocorticoid receptor with the c-*fos*-2 transcript was the mechanism proposed to explain the observed changes. Here we too show that the proportion between both c-*fos *transcript variants changes rapidly and transiently upon the stimulation by serum of NIH 3T3 fibroblasts in quiescence. Our data indicate that differential rates of synthesis determine the large up-regulation of spliced c-*fos *relative to c-*fos*-2 level at the early stage of the serum response. However, destabilization of the newly synthesized c-*fos *message, possibly as it is being translated, contributes to completing the response and restoring the ~50% yield (percentage of total transcript molecules) quantitated in confluent cultures. This temporal sequence of events further supports the idea that the alternative processing of the primary c-*fos *transcript might be of physiological relevance, given that a relatively short extension of c-*fos *expression is sufficient for fibroblasts to manifest properties of cell transformation [28].

Translation of the c-*fos*-2 transcript predicts the synthesis of a truncated protein lacking 211 aa at the carboxyl-terminus. We partially purified by immunoaffinity chromatography a c-Fos protein (verified by Western blot) with an apparent molecular weight of 23-kDa as judged by SDS-polyacrylamide gel electrophoresis. This finding suggests that the truncated variant of c-Fos is expressed in NIH 3T3 cells. The truncation would occur after the conserved basic domain for DNA binding but prior to the adjacent leucine zipper for dimerization (Fig. 8). Therefore, the truncated c-Fos should be inactive for transformation and transactivation since the functional integrity of the bZIP motif is required for these activities [29]. Moreover, the truncated c-Fos (unable to heterodimerize) should not exhibit antagonistic functional properties with the canonical longer counterpart, as in the case of ΔfosB [9-11]. On the other hand, however, the truncated c-Fos species might gain in stability as predicted by the loss of the main destabilizing activity residing at the C-terminal region [30,31].

What might the biological significance of the above-referred truncated variant of c-Fos protein be? One could speculate with the idea that the truncated species is just the side effect of the c-*fos*-2 translation occurring or tolerated in the cells. Nonetheless, a more active role in regulating gene expression could also be envisaged, since the truncated c-Fos would retain the DNA binding domain (Fig. 8). Two pathways have been described for binding dimeric proteins to DNA. In the dimer pathway, proteins first form dimers and then go on to bind DNA. Along the monomer pathway, a monomer•DNA complex is formed first, followed by recruitment of the second monomer to form the final complex. A growing number of dimeric DNA-binding proteins are able to form complexes with DNA via a monomer pathway. In fact, the monomer pathway is considered the best option for a faster and more specific formation of the final complex ([32] and references herein). Although Fos and Jun monomer•DNA complexes are difficult to observe, particularly in gel retardation experiments (e.g. see [33]), recent stopped-flow fluorescence studies on the kinetics of Fos•Jun•DNA complex formation indicate that both protein monomers bind DNA sequentially and assemble their dimerization interface while interacting with DNA [32]. These data allow us to speculate with the possibility [34] that even low levels of the truncated c-Fos variant might influence AP-1 mediated gene expression through the formation of non-productive monomer•DNA intermediates at specific DNA target sites. Additional experiments would be required to support such a notion.

## Conclusion

Here we provide the first absolute (molecule number) quantitative analysis of alternative spliced transcript isoforms derived from the c-*fos *precursor RNA. We confirm that intron 3 remains in the transcript population derived from c-*fos*. We demonstrate that this transcript (here referred as c-*fos*-2) is ubiquitously synthesized, either *in vivo *(various mouse-tissues, gestational-ages) or *in vitro *(different murine cell lines), in amounts that are higher or similar to those of mRNAs coding for other Fos family members. Changes in the synthesis and/or degradation rates of both isoforms (the canonical spliced c-*fos *mRNA and the c-*fos*-2 variant) indicate a co-ordinated fine-tune of transcript molecules, supporting the notion that the alternative processing of the c-*fos *precursor RNA might be physiologically relevant. Moreover, translation of the c-*fos*-2 transcript predicts the synthesis of a truncated c-Fos protein. We detected a c-Fos immunoreactive species corresponding in mobility to the predicted truncated protein.

## Methods

### Cell culture and treatments

NIH 3T3 (ATCC: CRL-1658) and Hepa1-6 (ATCC: CRL-1830) cells were cultured in Dulbecco's modified Eagle medium (DMEM) supplemented with 10% calf serum (CS) or foetal bovine serum, respectively. For mRNA decay quantitation, cells were seeded into 75 cm^2 ^flask at a density of 3 × 10^6 ^cells/flask and cultivated for 48 h before the addition of 10 μg/ml actinomycin D (AmD). At different times thereafter, cells were scraped, washed once with phosphate-buffered saline, and immediately frozen in liquid nitrogen for total RNA purification. For serum stimulation, NIH 3T3 cells were made quiescent by 48 h incubation in DMEM with 0.5% CS. Cultures were then stimulated by re-feeding quiescent cells with 15% CS. Cells were incubated further and collected at different times as for AmD treatment. Data presented are means of at least two independent cell cultures.

### Primer design

Primers directed against different regions of the c-*fos *and *fosB *genes (Fig. 1A) were designed with the Oligo 6.1 software (Molecular Biology Insights), as detailed in [35]. Primer positions (Fig. 1B) were chosen to detect intron sequences in the transcript population derived from c-*fos*. For comparison, primers for quantitation of the two well-defined forms of *fosB *transcript were also designed [9-11]. To obtain high specificity and performance, primers were required to have high Tm (≥82°C), optimal 3'-ΔG (≥ - 3 kcal/mol) value, and to be hairpin and duplex free. All primer pairs produced amplicons of the predicted size (Fig. 1C). All PCR products were further verified by nucleotide sequencing (ABI 377 DNA sequencer).

### RNA preparations and reverse transcription

Total RNA was extracted by using Tri-Reagent™ (Sigma) according to the manufacturer's protocol. Contaminating genomic DNA was removed by DNase I (Ambion). RNA quality was checked electrophoretically and quantitation was made spectrophotometrically. Lack of DNA contamination was confirmed by PCR amplification of RNA samples without previous cDNA synthesis. The standard RNA was synthesized *in vitro *from a laboratory-engineered DNA fragment containing a T7 polymerase-binding site, by means of a commercial RNA transcription kit (Stratagene). cDNA was generated from 2 μg of total RNA from each sample, using the M-MLV reverse transcriptase (Life Technologies) and random hexamers (Invitrogen).

### Real-time PCR

Real-time PCR reactions were performed in quadruplicate by using 50 ng of cDNA template, 0.3 μM of each primer (Fig. 1), 3 mM MgCl_2_, 250 μM of each dNTP, 0.75 units of Platinum Taq DNA polymerase, and 1:100000 SYBR Green I dye (Roche) in a volume of 25 μl. Reactions were analyzed on an iCycler iQ Real-Time PCR System (BioRad). Cycling conditions were as follows: 2 min at 95°C for the Platinum Taq activation and 40 cycles for the melting (15 s, 95°C) and annealing/extension (30 s, 70°C) steps. Replicate PCR reactions generated highly reproducible results with SEM <10% of the mean (<1% for threshold cycle) [see Additional file 5]. No primer-dimers were detected. Primers used for absolute quantitation of c-*fos *and *fosB *transcripts showed optimal (~100%) PCR efficiencies in the range of 20 to 2 × 10^5 ^pg of total RNA input with high linearity (r > 0.99) [see Additional file 6]. An absolute calibration curve was constructed with an external standard in the range of 10^2 ^to 10^9 ^RNA molecules [see Additional file 5]. The number of transcript molecules was calculated from the linear regression of the standard curve, as described previously [16] and exemplified in Additional file 5. The amount of c-*fos *mRNA molecules lacking intron 3 was estimated by subtracting the molecules with intron 3 (determined with E3U-I3L) from the total molecule number (determined with E3U-E4L). Similarly, the amount of mRNA molecules coding for ΔFosB was estimated by subtracting the molecules for the full-length FosB (determined with I4U-I4L) from the total molecule number (determined with E4U-E5L) (Fig. 1).

### Immunoaffinity purification of c-Fos proteins

Rabbit polyclonal Ab (Calbiochem, PC05) against the N-terminal region (aa 4–17) shared by the full-length and the putative truncated c-Fos protein were coupled to cyanogen bromide (CNBr)-activated agarose (Sigma C9142) following the manufacturer's recommendations. NIH 3T3 cells were cultured as described above. The accumulation of c-Fos proteins was promoted by 30 min serum stimulation. The cells were harvested and then lysed in 150 μl of buffer A (50 mM Tris-HCl pH7.4, 150 mM NaCl, 0.03% Tween 20, 0.4 mM Na_3_VO_4_, 0.4 mM EDTA, 10 mM NaF, 10 mM sodium pyrophosphate, 10 μg/ml aprotinin, 10 μg/ml leupeptin, and 1 mM phenylmethylsulfonyl fluoride), by using a polypropylene pestle for microcentrifuge tubes mounted in a cordless motor and 4 cycles of freezing in liquid nitrogen and thawing at room temperature. The cell lysate was centrifuged at 16000 × *g *for 10 min at 4°C. The resulting supernatant (crude extract) was applied to the immunoaffinity column, which was then washed extensively with buffer A. The bound proteins were eluted with two volumes of 100 mM glycine-HCl (pH 2.8). The eluate from the immunoaffinity column was neutralized with NaOH for Western blot analysis as described below.

### Western blotting

Proteins were separated on 12% sodium dodecyl sulfate (SDS)-polyacrylamide gel and transferred onto Hybond-P PVDF membrane (Amersham Biosciences) by electroblotting. Membranes were blocked with 2% non-fat milk powder in TTBS (25 mM Tris-HCl pH 7.6, 150 mM NaCl, 0.03% Tween-20) for 4–5 h at room temperature and then incubated overnight with 1:250 of primary Ab (Anti-c-Fos, Calbiochem PC05) in blocking buffer at 4°C. After washing the blots with blocking buffer, the membranes were incubated with 1:4000 of a secondary anti-rabbit IgG Ab (Sigma, A9169) to which horseradish peroxidase had been covalently coupled. Blots were developed using the ECL-Plus kit (Amersham Biosciences) following the manufacturer's instructions. The membranes were stripped by washing 30 min in stripping buffer (0.2 M glycine pH2.5, 0.1% SDS) at room temperature and reprobed with 1:2000 of anti-GAPDH Ab (Santa Cruz Biotechnology, sc-25778) for control loading.

## Abbreviations

AP-1, activator protein-1; bZIP, basic leucine-zipper; RT, reverse transcription; PCR, polymerase chain reaction; DMEM, Dulbecco's modified Eagle medium; CS, calf serum; AmD, actinomycin D; Cx, cycloheximide; aa, amino acid(s); Ab, antibody(ies); nt, nucleotide(s); DNase, deoxyribonuclease; PTC, premature termination codon; kDa, kilodalton(s)

## Competing interests

The author(s) declares that there are no competing interests.

## Authors' contributions

JJ carried out the purification of c-Fos proteins and CAF-A the absolute quantification of transcripts. In addition, both participated in the rest of the experiments and JJ helped to draft the manuscript. MJP-A participated in the determination of transcript half-lives and provided some cell culture samples. CP supervised the study design, participated in data analysis and drafted the manuscript. All the authors read and approved the final manuscript.

## Supplementary Material

Additional file 1 Empirical and theoretical decay of c-fos transcript. Various theoretical decays were generated under the assumption that the c-*fos*-2 transcript is merely a splicing intermediate and therefore the molecule number of c-*fos *might increase on behalf of c-*fos*-2 in a time-dependent manner in the presence of the transcription inhibitor AmD. Theoretical data are compared with experiment results shown in Fig. 4. Click here for file

Additional file 2 Confirmation that the ~23-kDa band detected by Western blot is not the primary Ab light chain. The E-30 eluate was subjected to inmunoblot analysis as described in Fig. 8. The ~23-kDa band was not detected when using only the secondary Ab, excluding the possibility that this band might be anti-cFos Ab light chains leaching off the immunoaffinity column. Click here for file

Additional file 3 Confirmation that the ~23-kDa band detected by Western blot is not a non-specific protein co-purified with the rabbit anti-cFos Ab. c-Fos proteins were partially purified by immunoaffinity chromatography as described under "Methods" for the experiment in Fig. 8. The exception was that the rabbit polyclonal anti-cFos Ab (Calbiochem, PC05) was coupled to N-hydroxysuccinimide (NHS)-activated (instead of to CNBr-activated) Sepharose (Amersham Biosciences 28-903-28). Western blotting was as described under "Methods", except for the primary (mouse monoclonal anti-cFos; Calbiochem OP17) and secondary (anti-mouse IgG; Sigma A9917) Ab. The immunogen used to generate the mouse anti-cFos Ab was a synthetic peptide corresponding to aa residues 128–152 (translation of the c-*fos*-2 transcript predicts the synthesis of a truncated protein 169 aa long). For other details see the Fig. 8 legend. Click here for file

Additional file 4 Comparison between poly(A)^+ ^and overall RNA populations. Total RNAs from NIH 3T3 cells treated as in Fig. 4 were retrotranscribed with random hexamers (see "RNA preparations and reverse transcription" subsection of "Methods") or anchored oligo(dT)primer (5'-T20VN-3') (Invitrogen). A) Overall (solid symbols) versus poly(A)^+ ^(open symbols) decay rate of c-*fos*-2 (circles) and c-*fos *(triangles) transcripts, respectively. B) Starting amounts (100% at time 0 min) of c-*fos *and c-*fos*-2 transcripts. Click here for file

Additional file 5 Absolute standard curve used to calculate the number of copies of each experimental transcript per pg of total RNA. A) The absolute standard curve was prepared with the *in vitro *synthesized standard RNA. The standard RNA [16] was a 457-nt fragment identical in sequence (except for a 7-bp deletion) to the mouse *gapdh *transcript (from 316 to 779 position in GenBank sequence M32599). The concentration of the standard RNA was determined by measuring the optical density at 260 nm and converting the absorbance to the number of copies by using its molecular weight. Ten-fold serial dilutions from 10^9 ^to 10^2 ^RNA copies were prepared, retrotranscribed and amplified by real-time PCR. Primers for the amplification of the standard have been previously described [16]. These primers generate a 130 bp PCR product. The standard curve was constructed by plotting the log of starting RNA molecules versus the threshold cycle (Ct). The resulting standard curve is linear (r = 0.998) over 7 orders of magnitude. The efficiency (E) value is calculated from the slope of the standard curve equation, as E = 10^[-1/slope]^-1. The slope of the standard curve indicates that the standard is amplified with 99.8% efficiency. This standard curve was used to determine the number of copies of each experimental transcript, as exemplified for c-*fos*-2 in total RNA from mouse ovary. B) Ct values used in the quantitation of the c-*fos*-2 transcript in 4 out of the 8 adult mouse tissues analyzed in Fig. 2. Real-time PCR reactions were carried out in quadruplicate by using 50 ng of cDNA template. Click here for file

Additional file 6 Real-time PCR efficiency of experimental transcript amplification. Ten-fold serial dilutions from 2 × 10^5 ^to 2 × 10^1 ^pg of total RNA input were prepared, retrotranscribed and amplified by real-time PCR. RNA sample was a pool of available total RNA from culture cells. A plot of the log RNA dilution versus the Ct value was made for each pair of primers, as exemplified here for E3U-E4L and E3U-I3L primers. Investigated transcripts showed optimal real-time PCR efficiencies of ~100% with a very high linearity (r>0.99) over 4 orders of magnitude. Click here for file

Additional file 7 PCR products amplified with E1U-E2L primer pair. Agarose (1.5%) gel electrophoresis analysis of PCR products generated by E1U-E2L primer pair. Cycling conditions were as in Fig. 1. Genomic DNA was from mouse liver. For comparison, total RNA from NIH 3T3 cells and cDNA retrotranscribed from this RNA were amplified in parallel with genomic DNA. Click here for file
