# Supplementary material for: Alternative splicing of c-fos pre-mRNA: contribution of the rates of synthesis and degradation to the copy number of each transcript isoform and detection of a truncated c-Fos immunoreactive species
Source: BMC Mol Biol. 2007 Sep 21;8:83. doi: 10.1186/1471-2199-8-83 (PMC2098773; doi:10.1186/1471-2199-8-83)
Supplement: Additional file 1 — Empirical and theoretical decay of c-fos transcript. Various theoretical decays were generated under the assumption that the c-fos-2 transcript is merely a splicing intermediate and therefore the molecule number of c-fos might increase on behalf of c-fos-2 in a time-dependent manner in the presence of the transcription inhibitor AmD. Theoretical data are compared with experiment results shown in Fig. 4. [file 1471-2199-8-83-S1.doc]

| **Additional file 1 - Empirical and theoretical decays of *c-fos* transcript** | | | | | | | | | | | | | |
| --- | --- | --- | --- | --- | --- | --- | --- | --- | --- | --- | --- | --- | --- |
|  | | *c-fos* molecules/pg RNA | | | | | | | | | | | |
|  | |  | | | theoretical | | | | | | | | |
| time in AmD (min) | | empirical | | | case 1 | | | case 2 | | | case 3 | | |
| 0 |  |  | 12 | |  | 12 | |  | 12 | |  | 12 | |
| 15 |  |  | | 6.30 |  | | 1.21 |  | | 8.11 |  | 11.74 | |
| 30 |  |  | | 4.70 |  | | 0.04 |  | | 2.63 |  | | 6.00 |
| 60 |  |  | | 1.10 |  | | 0 |  | | 0.26 |  | | 1.55 |

Data are c-*fos* transcript molecules/pg of total RNA. Empirical data are those in Fig. 4 (NIH 3T3 cells; t_1/2_=17.9 min). Theoretical numbers were generated under the assumption that c-*fos*-2 transcript is merely a splicing intermediate and therefore the molecule number of c-*fos* might increase on behalf of c-*fos*-2 in a time-dependent manner in the presence of the transcription inhibitor AmD. Theoretical case 1 supposes an actual t_1/2_ for the spliced c-*fos* of 3 min, case 2 a t_1/2_ of 9 min, and case 3 a t_1/2_ of 15 min.
