# Supplementary figures and images for: Alternative splicing of c-fos pre-mRNA: contribution of the rates of synthesis and degradation to the copy number of each transcript isoform and detection of a truncated c-Fos immunoreactive species
Source: BMC Mol Biol. 2007 Sep 21;8:83. doi: 10.1186/1471-2199-8-83 (PMC2098773; doi:10.1186/1471-2199-8-83)

## Slide 1
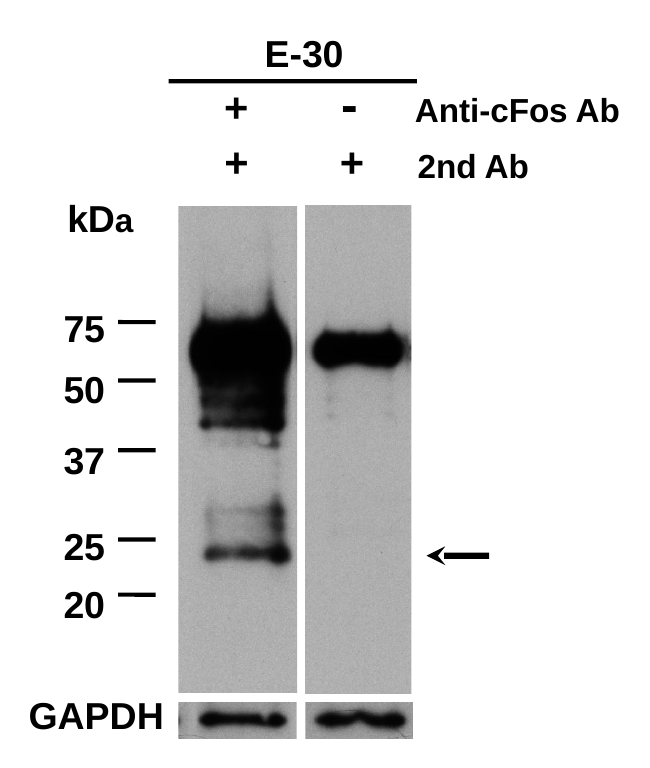

E-30
-
+
Anti-cFos Ab
+
+
2nd Ab
kDa
75
50
37
25
20
GAPDH

Supplement: Additional file 2 — Confirmation that the ~23-kDa band detected by Western blot is not the primary Ab light chain. The E-30 eluate was subjected to inmunoblot analysis as described in Fig. 8. The ~23-kDa band was not detected when using only the secondary Ab, excluding the possibility that this band might be anti-cFos Ab light chains leaching off the immunoaffinity column. [file 1471-2199-8-83-S2.ppt]

## Slide 1
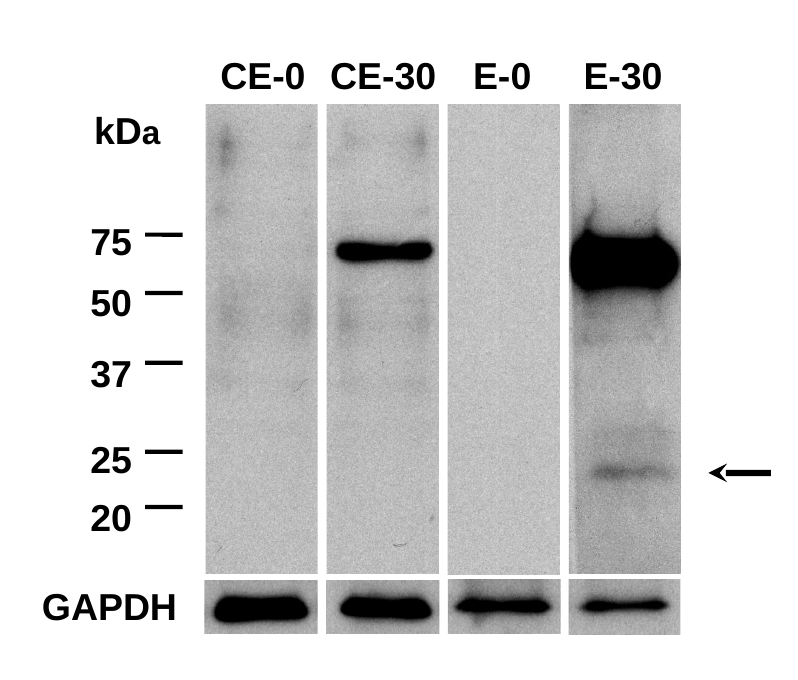

CE-0
CE-30
E-0
E-30
kDa
75
50
37
25
20
GAPDH

Supplement: Additional file 3 — Confirmation that the ~23-kDa band detected by Western blot is not a non-specific protein co-purified with the rabbit anti-cFos Ab. c-Fos proteins were partially purified by immunoaffinity chromatography as described under "Methods" for the experiment in Fig. 8. The exception was that the rabbit polyclonal anti-cFos Ab (Calbiochem, PC05) was coupled to N-hydroxysuccinimide (NHS)-activated (instead of to CNBr-activated) Sepharose (Amersham Biosciences 28-903-28). Western blotting was as described under "Methods", except for the primary (mouse monoclonal anti-cFos; Calbiochem OP17) and secondary (anti-mouse IgG; Sigma A9917) Ab. The immunogen used to generate the mouse anti-cFos Ab was a synthetic peptide corresponding to aa residues 128–152 (translation of the c-fos-2 transcript predicts the synthesis of a truncated protein 169 aa long). For other details see the Fig. 8 legend. [file 1471-2199-8-83-S3.ppt]

## Slide 1
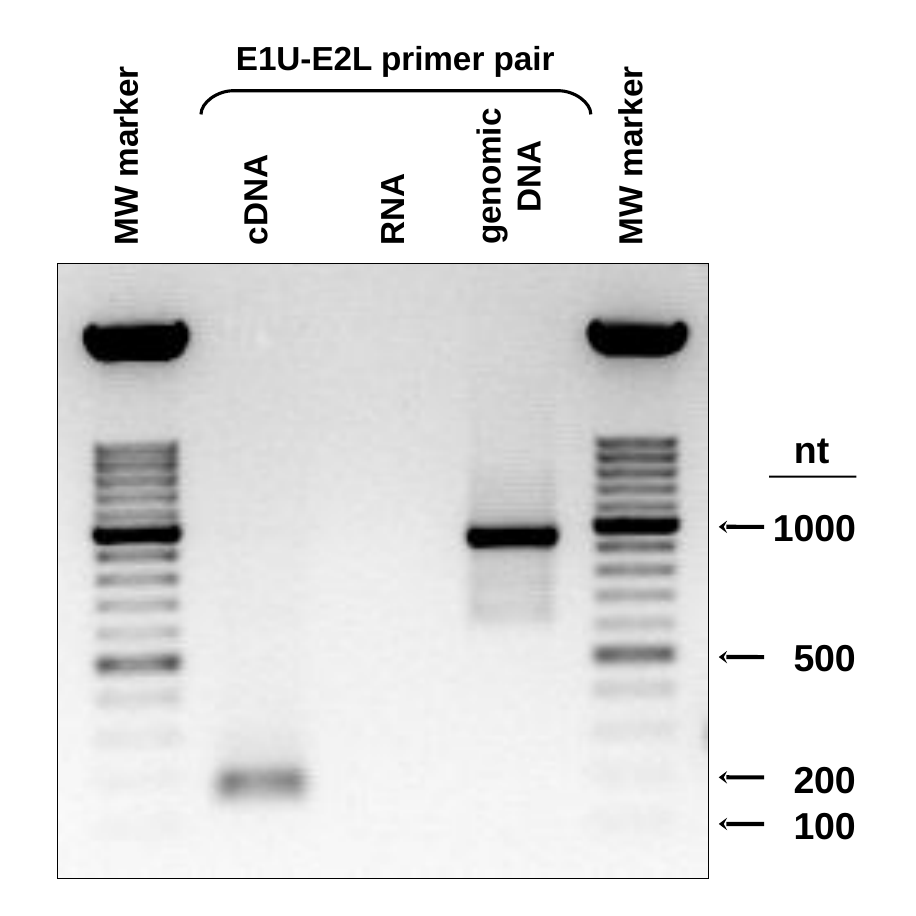

E1U-E2L primer pair
MW marker
MW marker
genomic
DNA
cDNA
RNA
nt
1000
500
200
100

Supplement: Additional file 7 — PCR products amplified with E1U-E2L primer pair. Agarose (1.5%) gel electrophoresis analysis of PCR products generated by E1U-E2L primer pair. Cycling conditions were as in Fig. 1. Genomic DNA was from mouse liver. For comparison, total RNA from NIH 3T3 cells and cDNA retrotranscribed from this RNA were amplified in parallel with genomic DNA. [file 1471-2199-8-83-S7.ppt]
