# Supplementary material for: Alternative splicing of c-fos pre-mRNA: contribution of the rates of synthesis and degradation to the copy number of each transcript isoform and detection of a truncated c-Fos immunoreactive species
Source: BMC Mol Biol. 2007 Sep 21;8:83. doi: 10.1186/1471-2199-8-83 (PMC2098773; doi:10.1186/1471-2199-8-83)
Supplement: Additional file 4 — Comparison between poly(A)+ and overall RNA populations. Total RNAs from NIH 3T3 cells treated as in Fig. 4 were retrotranscribed with random hexamers (see "RNA preparations and reverse transcription" subsection of "Methods") or anchored oligo(dT)primer (5'-T20VN-3') (Invitrogen). A) Overall (solid symbols) versus poly(A)+ (open symbols) decay rate of c-fos-2 (circles) and c-fos (triangles) transcripts, respectively. B) Starting amounts (100% at time 0 min) of c-fos and c-fos-2 transcripts. [file 1471-2199-8-83-S4.ppt]

## Slide 1
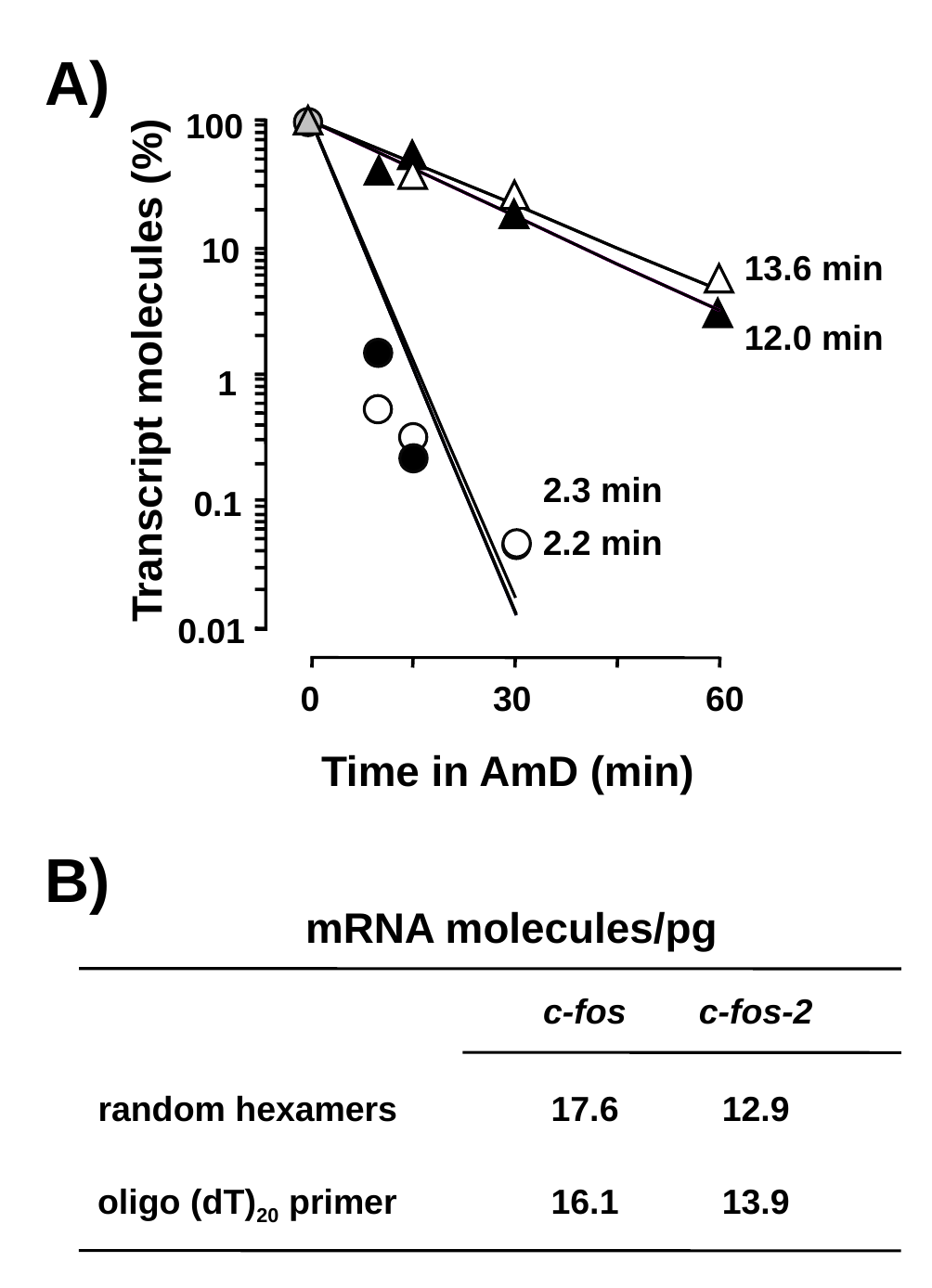

A)
100
10
13.6 min
12.0 min
Transcript molecules (%)
1
2.3 min
0.1
2.2 min
0.01
0
30
60
Time in AmD (min)
B)
mRNA molecules/pg
c-fos
c-fos-2
random hexamers
17.6
12.9
oligo (dT)20 primer
16.1
13.9
