# Supplementary material for: Alternative splicing of c-fos pre-mRNA: contribution of the rates of synthesis and degradation to the copy number of each transcript isoform and detection of a truncated c-Fos immunoreactive species
Source: BMC Mol Biol. 2007 Sep 21;8:83. doi: 10.1186/1471-2199-8-83 (PMC2098773; doi:10.1186/1471-2199-8-83)
Supplement: Additional file 5 — Absolute standard curve used to calculate the number of copies of each experimental transcript per pg of total RNA. A) The absolute standard curve was prepared with the in vitro synthesized standard RNA. The standard RNA [16] was a 457-nt fragment identical in sequence (except for a 7-bp deletion) to the mouse gapdh transcript (from 316 to 779 position in GenBank sequence M32599). The concentration of the standard RNA was determined by measuring the optical density at 260 nm and converting the absorbance to the number of copies by using its molecular weight. Ten-fold serial dilutions from 109 to 102 RNA copies were prepared, retrotranscribed and amplified by real-time PCR. Primers for the amplification of the standard have been previously described [16]. These primers generate a 130 bp PCR product. The standard curve was constructed by plotting the log of starting RNA molecules versus the threshold cycle (Ct). The resulting standard curve is linear (r = 0.998) over 7 orders of magnitude. The efficiency (E) value is calculated from the slope of the standard curve equation, as E = 10[-1/slope]-1. The slope of the standard curve indicates that the standard is amplified with 99.8% efficiency. This standard curve was used to determine the number of copies of each experimental transcript, as exemplified for c-fos-2 in total RNA from mouse ovary. B) Ct values used in the quantitation of the c-fos-2 transcript in 4 out of the 8 adult mouse tissues analyzed in Fig. 2. Real-time PCR reactions were carried out in quadruplicate by using 50 ng of cDNA template. [file 1471-2199-8-83-S5.ppt]

## Slide 1
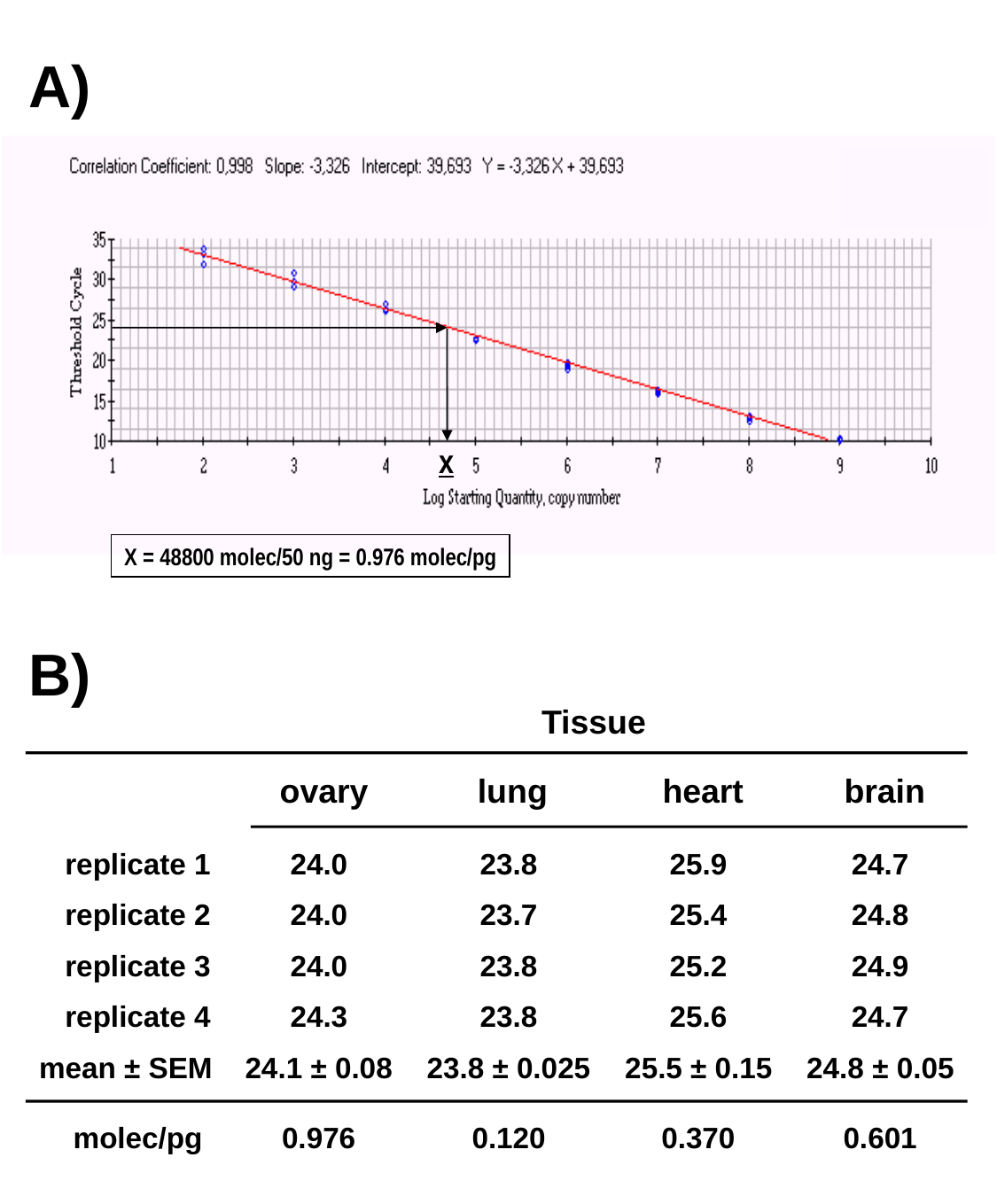

A)
x
X = 48800 molec/50 ng = 0.976 molec/pg
B)
Tissue
ovary
lung
heart
brain
replicate 1
24.0
23.8
25.9
24.7
replicate 2
24.0
23.7
25.4
24.8
replicate 3
24.0
23.8
25.2
24.9
replicate 4
24.3
23.8
25.6
24.7
mean ± SEM
24.1 ± 0.08
23.8 ± 0.025
25.5 ± 0.15
24.8 ± 0.05
molec/pg
0.976
0.120
0.370
0.601
