# Supplementary material for: Alternative splicing of c-fos pre-mRNA: contribution of the rates of synthesis and degradation to the copy number of each transcript isoform and detection of a truncated c-Fos immunoreactive species
Source: BMC Mol Biol. 2007 Sep 21;8:83. doi: 10.1186/1471-2199-8-83 (PMC2098773; doi:10.1186/1471-2199-8-83)
Supplement: Additional file 6 — Real-time PCR efficiency of experimental transcript amplification. Ten-fold serial dilutions from 2 × 10^5^ to 2 × 101 pg of total RNA input were prepared, retrotranscribed and amplified by real-time PCR. RNA sample was a pool of available total RNA from culture cells. A plot of the log RNA dilution versus the Ct value was made for each pair of primers, as exemplified here for E3U-E4L and E3U-I3L primers. Investigated transcripts showed optimal real-time PCR efficiencies of ~100% with a very high linearity (r>0.99) over 4 orders of magnitude. [file 1471-2199-8-83-S6.ppt]

## Slide 1
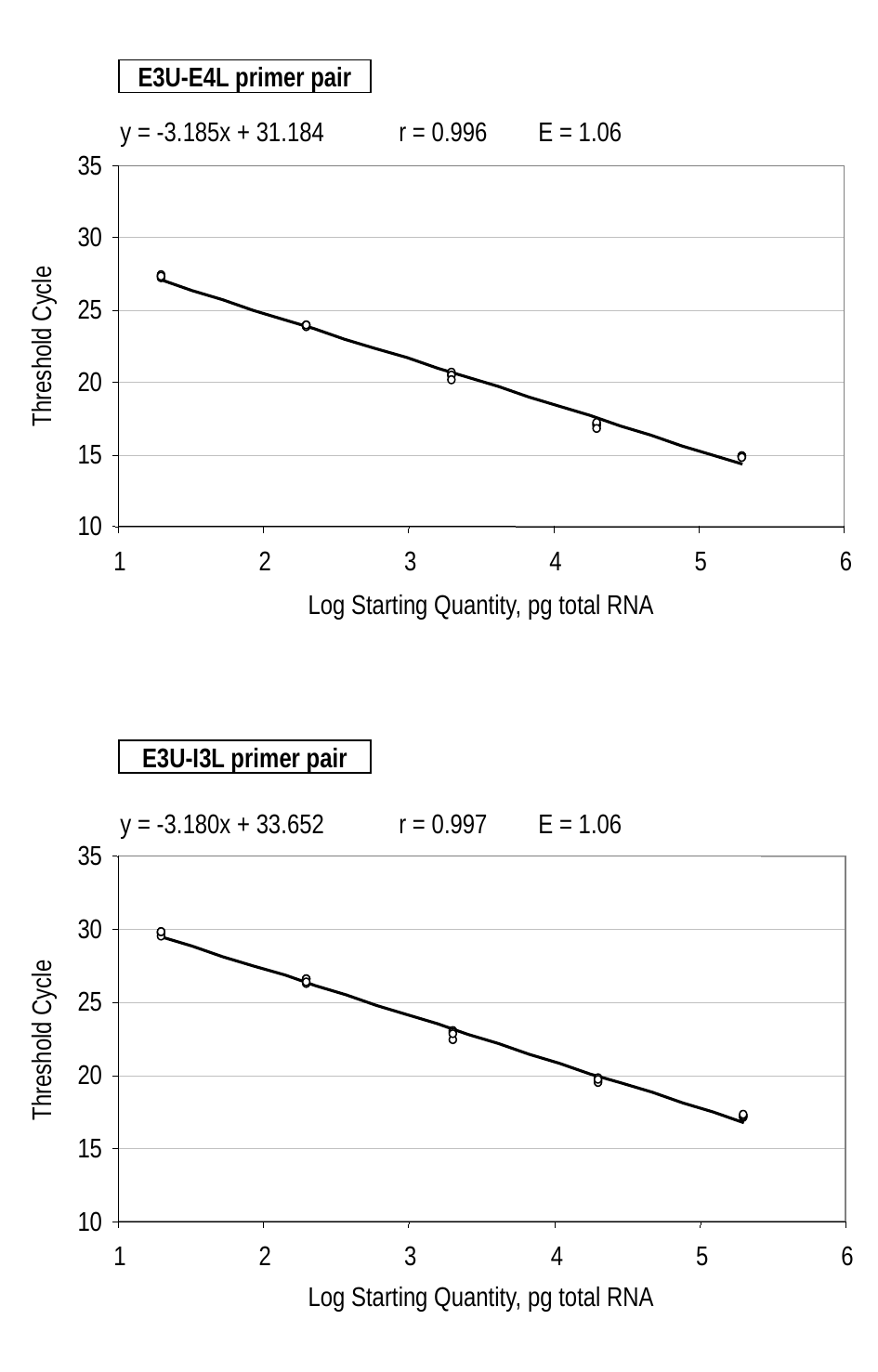

E3U-E4L primer pair
y = -3.185x + 31.184	r = 0.996	E = 1.06
35
30
25
20
15
10
1
2
3
4
5
6
Threshold Cycle
Log Starting Quantity, pg total RNA
E3U-I3L primer pair
y = -3.180x + 33.652	r = 0.997	E = 1.06
35
30
25
20
15
10
1
2
3
4
5
6
Threshold Cycle
Log Starting Quantity, pg total RNA
